# Teleconsultation at a public ophthalmic teaching hospital during the COVID-19 pandemic

**DOI:** 10.5935/0004-2749.2021-0490

**Published:** 2022-10-19

**Authors:** Camila Ribeiro Koch, Caio Cezar Andrade Veiga, Renata Fahl, Sheila Moreno Halla, Newton Kara Junior, Milton Ruiz Alves

**Affiliations:** 1 Universidade de São Paulo, São Paulo, SP, Brazil; 2 Hospital Humberto Castro Lima, Salvador, Brazil

**Keywords:** COVID-19, Telemedicine, Pandemics, Remote consultation, Eye diseases/diagnosis, Hospitals, public, COVID-19, Telemedicina, Pandemias, Consulta remota, Oftalmopatias/diagnóstico, Hospitais públicos

## Abstract

**Purpose:**

To analyze teleconsultation at a public ophthalmic teaching hospital during the COVID-19 pandemic in Brazil.

**Methods:**

Medical records of patients who requested ophthalmological teleconsultation between June 2020 and March 2021 were reviewed. The main outcomes included demographic data, eye disease symptoms, hypothesized diagnosis, and management. Moreover, the results of a satisfaction survey administered after the consultation were analyzed.

**Results:**

Medical records of a total of 161 patients were reviewed. The mean age was 45.98 ± 17.57 (8-90) years, and most were women (113, 70.20%). Only 57 (35.60%) of the patients had made previous follow-up visits to the hospital. The most frequent reason for consultation was the need for a new eyeglass prescription (73, 45.34%), followed by dry eye symptoms (16, 9.93%) and pterygium (13, 8.07%). Other reasons were the monitoring of previously diagnosed eye diseases, such as glaucoma, retinopathies, strabismus, and keratoconus. Regarding the satisfaction survey, 151 (93.78%) patients answered the online questionnaire. Most reported that they were satisfied with the teleconsultation (94.03%) and would participate in a future teleconsultation (90.06%).

**Conclusion:**

Teleconsultation could be widely used to assist patients in public ophthalmology healthcare and teaching hospitals. Even though new eyeglass prescriptions are a frequent reason for ophthalmological appointments, patients tend to be satisfied with teleconsultation, as it also provides guidance.

## Abstract (pt)

**Objetivo:**

Analisar a teleconsulta em um hospital público de ensino oftalmológico, durante o período da pandemia do COVID-19.

**Métodos:**

Foram revisados os registros médicos dos pacientes que solicitaram teleconsulta oftalmológica, no período de Junho de 2020 a Março de 2021. Os resultados incluem dados demográficos, sintomas de queixas oculares e hipóteses diagnósticas. Além disso, foram analisados dados da pesquisa de satisfação aplicada após cada teleconsulta.

**Resultados:**

Um total de 161 prontuários foram revisados. A idade média dos pacientes foi de 45.98 ± 17.57 (8 a 90) anos, a maioria mulheres, 113 (70,20%). Apenas 57 (35,60%) eram pacientes acompanhados no hospital previamente. A principal razão pela busca pela teleconsulta foi o erro refracional, 73 (45.43%), seguido de olho seco, 16 (9.93%), pterígio, 13 (8.07%). Outros motivos foram o acompanhamento de doenças prévias como glaucoma, retinopatias, miopia, estrabismo e ceratocone. Quanto a pesquisa de satisfação, 151(93,87%) pacientes responderam a pesquisa on-line. A maioria deles mostrou-se satisfeito com a teleconsulta (94.03%) e fariam uma nova teleconsulta (90.06%).

**Conclusão:**

A teleconsulta pode auxiliar a saúde pública em oftalmologia podendo ser utilizada em hospitais universitários. Embora o erro refracional tenha sido o motivo mais frequente nas consultas, os pacientes mostraram-se satisfeitos com essa modalidade de atendimento que serve como um serviço de orientação.

## INTRODUCTION

Teleconsultation expands healthcare by allowing physicians to screen for emergency issues, monitor chronic diseases, and follow-up on treatment plans and prescriptions^(1,2)^. In ophthalmology, telehealth enables increased screening for diseases such as diabetic retinopathy, retinopathy of prematurity, age-related macular degeneration, and glaucoma^(3,4)^. With the present coronavirus disease-2019 (COVID-19) pandemic, telehealth has become more accessible in various applications^(5)^, such as ophthalmology, a medical specialty with a high risk of COVID transmission during consultation considering the face-to-face proximity^(6)^.

Teleconsultation has a well-established capability to compensate for geographical barriers and can be readily accepted by the population^(3)^. Indeed, it is a common practice in countries such as France, India, and the United States^(1,7)^. In Brazil, although the waiting period for a face-to-face ophthalmological consultation in the public health system may exceed 1 year, teleconsultation is still not widespread^(2,8,9,10)^. Telehealth should be considered for routine medical consultations. During lockdowns because of the COVID-19 pandemic, an eye hospital in Singapore reported that 80% of the appointments were postponed or rescheduled, leaving many patients without access to eye care^(11,12)^. Moreover, Host et al.^(13)^ described a high level of satisfaction with teleconsultation in an Australian hospital because participants reported saving time and money through virtual appointments.

The COVID-19 pandemic has restricted access to healthcare systems because of the need for social distancing, and health services have employed teleconsultation to assist the population. This practice reduces the risk of exposure for professionals and patients in addition to improving healthcare accessibility in regions^(11,14,15)^. In this context, this study aimed to describe the outcomes of the implementation of teleconsulting services in a Brazilian public ophthalmic teaching hospital during the COVID-19 pandemic and analyze patient satisfaction.

## METHODS

### Study population

In this observational, retrospective study, data of patients who requested teleconsultation in ophthalmology at the Hospital Humberto Castro Lima, Salvador, Brazil, between June 1, 2020, and March 31, 2021, were reviewed. This study followed the tenets of the Declaration of Helsinki and was approved by the Medical Institutional Review Board of Bahiana School of Medicine and Public Health, Salvador.

### Data collection

The data collected included each patient’s birth date, sex, reason for consultation, symptoms, main diagnosis, and management. Data from a satisfaction survey were also analyzed.

### Management of teleconsultation

Teleconsultation was offered to interested patients during the pandemic. Appointments were made by telephone or video call. Teleconsultation was conducted by video through a telephone offered by the hospital. The call was made in a private room, with only the consulting physician present, and thus did not pose a risk of COVID-19 exposure. The physician had the patient’s medical record on hand; thus, it was possible to identify whether the patient had already been followed up at the hospital. The consulting physician was an ophthalmologist from the fellowship program of Hospital Humberto Castro Lima, who was supervised by a preceptor. If there were problems with network instability, the appointment could be rescheduled.

During consultation, complete anamnesis was performed. Patients were asked about signs and symptoms, comorbidities, use of systemic medications and eye drops, ophthalmological diseases, and family ophthalmological history. If a pathology was diagnosed, a prescription was sent by email or text, and a detailed explanation was provided. If diagnosis was not possible or if the patient did not recover and needed additional examinations, a face-to-face appointment at the hospital was scheduled.

### Satisfaction survey

An ophthalmologist conducted a satisfaction survey in the days following the video appointment; the questionnaire was administered to the patients privately by telephone. Each respondent was asked whether he or she was satisfied with the teleconsultation, whether his or her problem was solved, whether there were any technical problems, and whether he or she would schedule a future teleconsultation.

## RESULTS

The medical records of 161 patients were analyzed. The mean age of the patients was 45.98 ± 17.57 (8-90) years, and most of the patients were women (113, 70.20%). Among the patients, 105 (65.22%) reported no previous eye disorders. Table 1 describes demographic data, diagnoses of previous ophthalmologic pathology, systemic diseases, and previous ophthalmologic surgeries. Moreover, 57 (35.60%) patients had already been consulted at the hospital previously.

**Table 1:** Demographic data and medical history of the patients

	Total n (%)		Total n (%)
Sex		Past medical history	
Male	48 (29.80%)	Ophthalmological	
Female	113 (70.20%)	None	105 (65.22%)
Age	45.98 ± 17.57	Glaucoma	12 (7.54%)
Follow-up		Strabismus	12 (7.54%)
New patient	104 (64.41%)	Diabetic retinopathy	7 (4.34%)
Followed	57 (35.59%)	High myopia	4 (2.48%)
Call type		Keratoconus	4 (2.48%)
Video	154 (95.65%	Previous ophthalmic surgery	
Audio	7 (4.35%)	None	119 (73.91%)
Past medical history		Phacoemulsification	24 (14.90%)
Systemic		Pterygium **excision**	12 (7.45%)
None	98 (60.86%)	Posterior vitrectomy	5 (3.10%)
Arterial hypertension	40 (24.84%)	Strabismus correction	2 (1.24%)
Diabetes	25 (15.52%	Glaucoma surgery	2 (1.24%)
Asthma	6 (3.72%)		
Sickle cell anemia	2 (1.24%)		
Lupus	1 (0.62%)		

### Main outcomes

The most common reason for teleconsultation was the need for a new eyeglass prescription (n = 71, 45.34%), followed by dry eye symptoms (n = 16, 9.93%), pterygium evaluation (n = 13, 8.07%), and monitoring of previously diagnosed diseases (n = 12, 7.44%) such as glaucoma, retinopathies, and keratoconus. Moreover, 9 (5.59%) patients sought the service to schedule surgery for strabismus, 8 (4.96%) to arrange an evaluation for cataract surgery, and 3 (1.86%) to arrange surgery for dermatochalasis. Only 3 (1.86%) patients had acute conditions, such as hordeolum and conjunctivitis.

**Figure 1:**
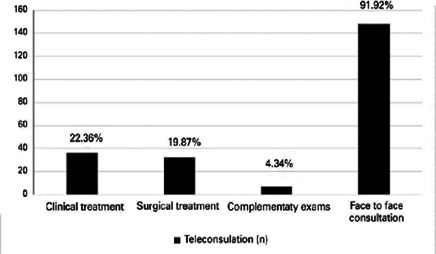
Outcomes of the medical referral.

Ophthalmologists prescribed lubricating eye drops to 33 (20.49%) patients and antibiotic eye drops to 3 (3.10%). In addition, 26 (16.14%) patients were referred for surgical evaluation, such as pterygium, cataract, and blepharoplasty surgery. Other medical tasks included scheduling a surgical procedure (n = 6, 3.72%), arranging a face-to-face ophthalmological examination (n = 5, 3.10%), and discussing preoperative results (n = 2, 1.24%) (Figure 1).

In-person appointments were suggested in 148 (91.92%) of the 161 appointments. All reassessments were performed by face-to-face consultation rather than teleconsultation.

### Satisfaction survey

Of the total sample, 151 (93.78%) patients answered the survey. A total of 142 (94.03%) were satisfied with the teleconsultation, and 136 (90.06%) would arrange a future teleconsultation. A total of 125 (82.78%) responded that their problems were solved. Only 5 (3.31%) reported technical problems, such as network instability (Table 2). In these cases, video calls were replaced with voice calls, and they were not rescheduled.

**Table 2:** Outcomes of the satisfaction survey

	Yes	No
Were you satisfied with the teleconsultation?	142 (94.03%)	9 (5.97%)
Did the teleconsultation solve your problem?	125 (82.78%)	26 (17.22%)
Was there a technical problem?	5 (3.31%)	146 (96.69%)
Would you conduct a future consultation by telemedicine?	136 (90.06%)	15 (9.94%)

## DISCUSSION

Teleconsultation, a form of interaction between doctors and patients, has become an important form of healthcare during the COVID-19 pandemic. During the pandemic, teleconsultation allowed social distancing, reducing the spread of infection^(1,5)^. Lockdowns forced health services to implement this modality quickly to meet the population’s demand. With the benefits of teleconsultation, discontinuing this practice would be a setback. The results of this study demonstrated the possibility of maintaining ophthalmological care through a public ophthalmological service in Brazil while maintaining strict social distancing precautions.

Bourdon et al.^(7)^ reported that hordeolum was the main pathology that motivated consultations, and 27% of the patients with this condition were referred for in-person appointments. This French study, unlike ours, was conducted at an emergency service, which may explain this divergence in the reasons behind telecon-sultation. Our results showed that refractive errors were the main motivation, and 91% of our patients needed in-person appointments. In both studies, most of the patients were female.

Most patients who sought teleconsultation had nonurgent complaints and oriented, whereas others were directed to face-to-face care as soon as possible. Given the social confinement, the presence of medical attention, even if it is distant, could reassure patients about their health condition, in addition to contributing to the continuity of care.

In our study, most cases of eyelid and ocular surface disorders were resolved by teleconsultations, as were most ophthalmological and preoperative examinations. In the case of refractive errors, corneal and lens disorders, or posterior segment diseases, physical consultation was suggested for most of the patients because our hospital does not yet have a remote office for a more detailed physical examination. Even though most of the cases were not emergencies, the population was confined during this pandemic period, and clarifications in these non-emergencies could settle down them.

Valpuesta Martin et al. ^(16)^ demonstrated that 93.8% of their patients were satisfied with a teleophthalmology-based screening program for diabetic retinopathy. This service involves retinographies of patients with diabetes in a health center, and the images are sent by a teleophthalmology system to ophthalmologists who report the examinations. Based on the classification of diabetic retinopathy, the patient may or may not be referred to face-to-face consultation.

Arntz et al.^(6)^ also found high patient satisfaction, in which 100% of their patients were satisfied, in a pilot study of teleophthalmology during the COVID-19 pandemic. The consultation was conducted by video calls by an ophthalmologist and lasted 30 min. Regardless of the high current level of satisfaction referred by patients, the use of advanced technology, high-definition images, and a good internet network can guarantee even greater success in teleconsultation. In this study, despite the high number of referrals for face-to-face consultation, most patients were satisfied with the virtual services provided through video calls, as shown by the results of the satisfaction survey after the teleconsultations. When asked if they would arrange a future teleconsultation, almost all of them answered yes (90%), confirming their satisfaction. Only seven participants reported technical problems during consultations; when these problems arose, voice calls were made for the consultations. Two of these patients were dissatisfied and would not arrange a future appointment in this modality. We believe that if our service had an equipped remote ophthalmic office with guidance to primary care professionals, as in other services^(9)^, the number of face-to-face consultations would be reduced with greater resolution.

Teleconsultation is not a widespread practice in the Brazilian healthcare system, even though Brazil can apply telemedicine because of its large geographical area, presence of isolated communities, and unequal distribution of medical resources. Remote units with telepresence systems have been successfully used for ophthalmological consultation in distant cities in the southern part of the country. Specifically, ophthalmologists have used digital refractometers and visual acuity screens in basic health units for remote consultations^(9)^. These technologies enabled clinicians to measure refractive error. In addition, the program reduced the number of face-to-face consultations and identified patients with ophthalmic emergencies, prioritizing these appointments.

Public policies to promote teleconsultation in ophthalmology cannot only bring benefits during the COVID-19 pandemic period but also reduce the influx of patients at the hospital through prior screening and prioritization of care. Thus, teleconsulting is an instrument that guarantees universal access to healthcare, and other public health services should take the opportunity to encourage it. The results of this study suggest that teleconsultation could be used in healthcare even after the pandemic and that most patients were satisfied with this modality of care.
